# Supplementary figures and images for: Simultaneous epigenetic perturbation and genome imaging reveal distinct roles of H3K9me3 in chromatin architecture and transcription
Source: Genome Biol. 2020 Dec 8;21:296. doi: 10.1186/s13059-020-02201-1 (PMC7722448; doi:10.1186/s13059-020-02201-1)

**A**

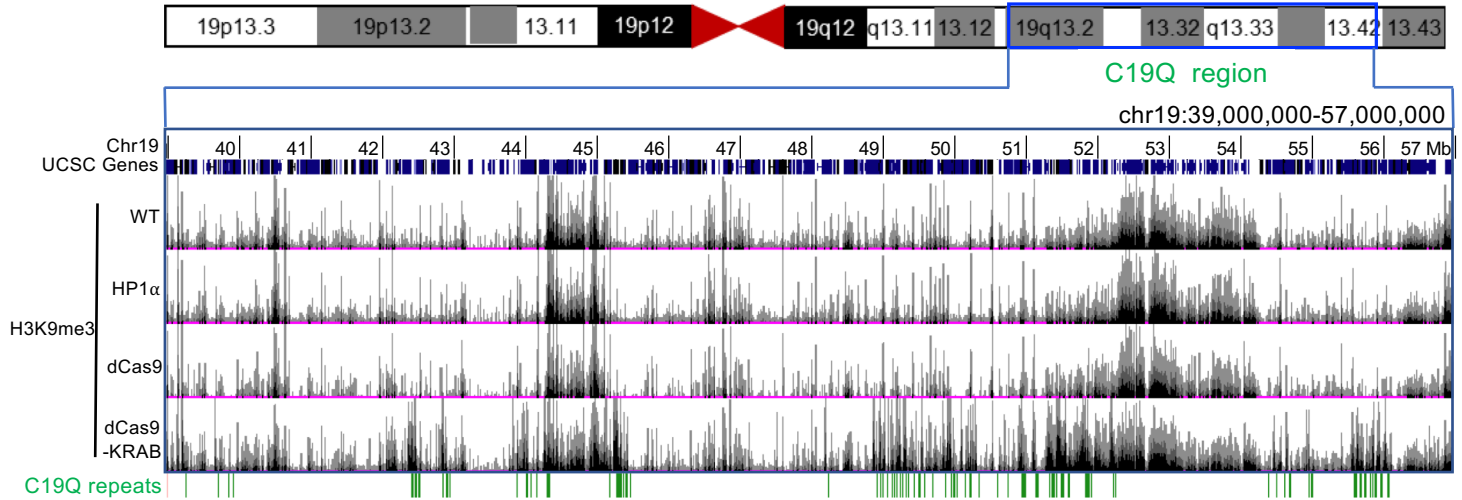

**B**

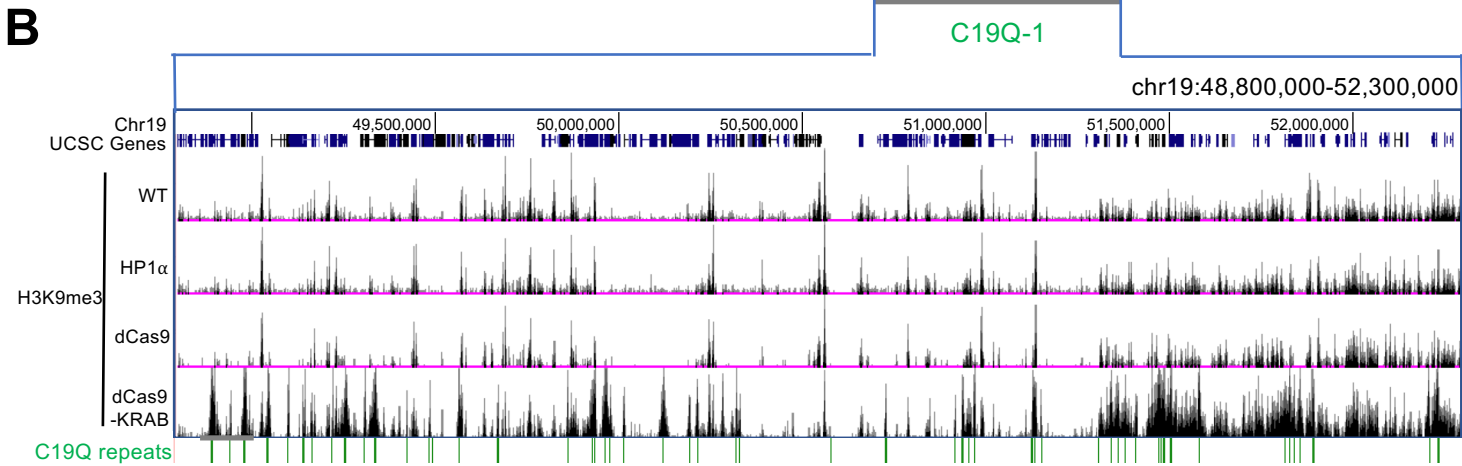

Supplement: Supplementary file 1 — Additional file 1: Figure S1. H3K9 trimethylation of C19Q regions mediated by the EpiGo-KRAB system. (A) H3K9me3 states of C19Q region in the cell lines of U2OS wildtype (WT), U2OS-HP1α-HaloTag (HP1α), EpiGo-Control (dCas9) or EpiGo-KRAB (dCas9-KRAB). ChIP-seq of H3K9me3 was performed in these four cell lines. C19Q repeats shown the C19Q target sites. (B) C19Q-1 region is chosen to show the difference of H3K9me3 state between U2OS wildtype (WT), U2OS-HP1α-HaloTag (HP1α), EpiGo-Control (dCas9) or EpiGo-KRAB (dCas9-KRAB) cell lines. C19Q repeats shown the C19Q target sites. Figure S2. Negative correlation of H3K9 trimethylation and active transcription. The H3K9me3 state and RNA levels of 30-40 Mb regions in chromosome 1-6 were shown in A-F respectively. The ChIP-seq of H3K9me3 and RNA-seq were performed on U2OS-EpiGo-Control cell lines. Figure S3. The effect of target distance on gene expression. (A) Plot showing the relationship between distance (CRISPR to the nearest TSS) and gene expression fold change. (B) Plot showing the relationship between distance (CRISPR to the nearest TSS) and promoter H3K4me3 enrichment change. (C) Plot showing the relationship between distance (CRISPR to the nearest TSS) and promoter H3K27ac enrichment change. [file 13059_2020_2201_MOESM1_ESM.zip › Additional file 1/Figure S1.pdf]

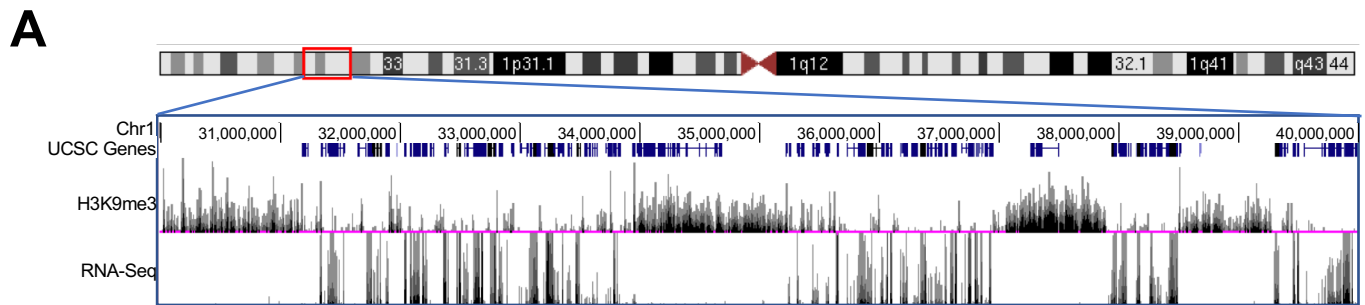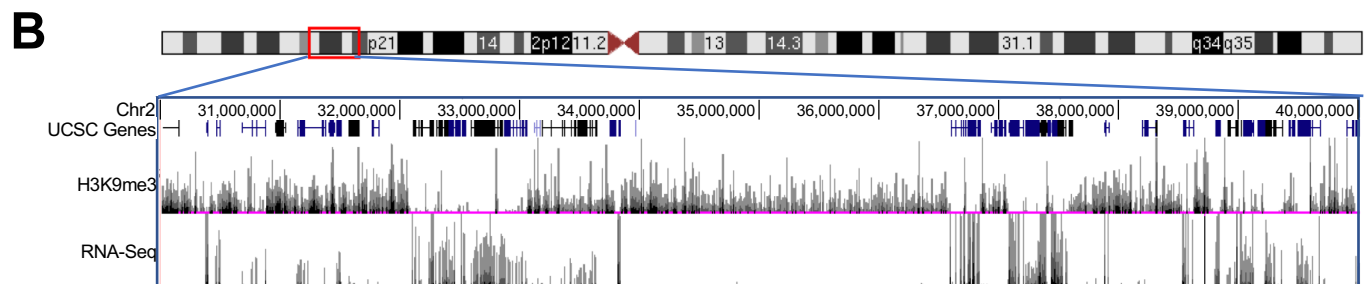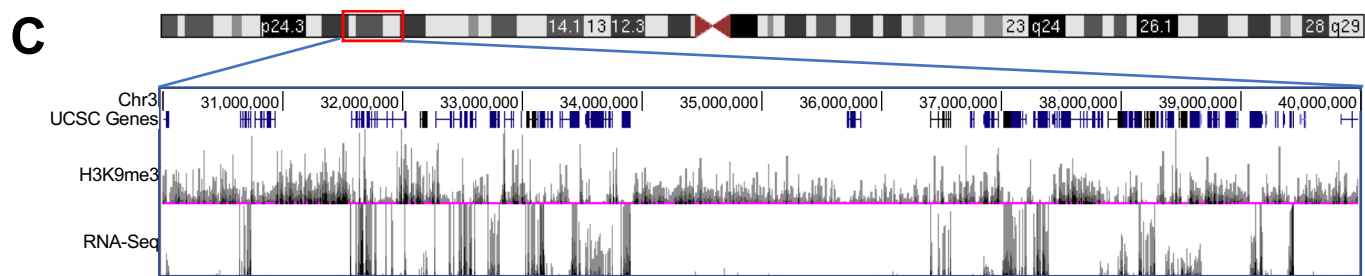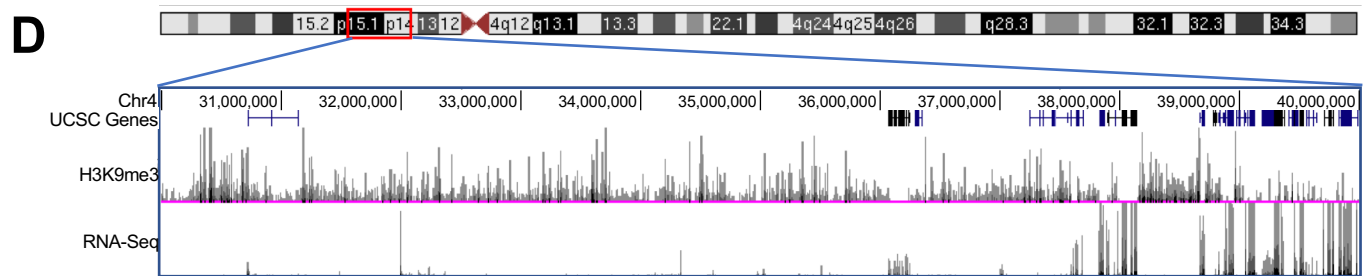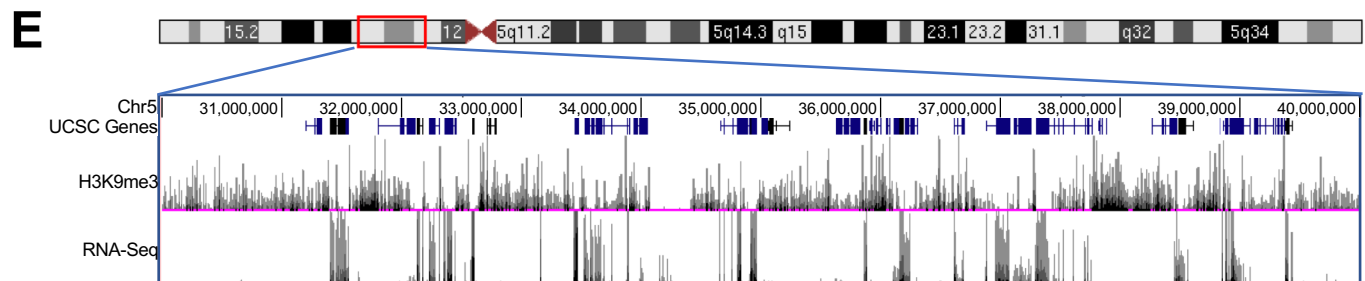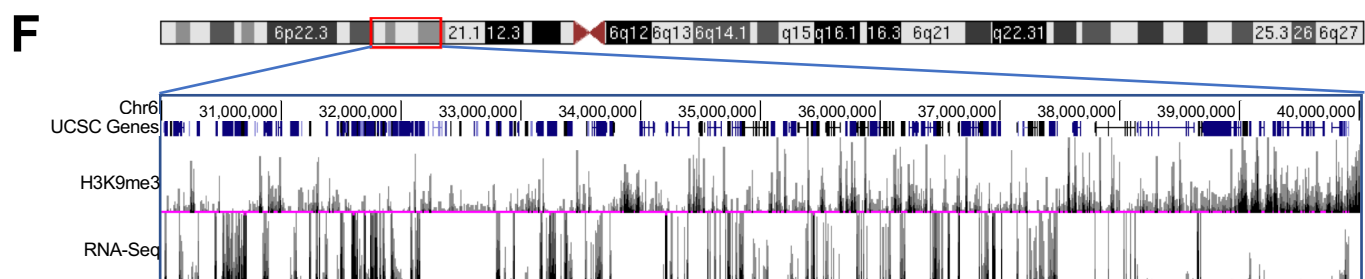

Supplement: Supplementary file 1 — Additional file 1: Figure S1. H3K9 trimethylation of C19Q regions mediated by the EpiGo-KRAB system. (A) H3K9me3 states of C19Q region in the cell lines of U2OS wildtype (WT), U2OS-HP1α-HaloTag (HP1α), EpiGo-Control (dCas9) or EpiGo-KRAB (dCas9-KRAB). ChIP-seq of H3K9me3 was performed in these four cell lines. C19Q repeats shown the C19Q target sites. (B) C19Q-1 region is chosen to show the difference of H3K9me3 state between U2OS wildtype (WT), U2OS-HP1α-HaloTag (HP1α), EpiGo-Control (dCas9) or EpiGo-KRAB (dCas9-KRAB) cell lines. C19Q repeats shown the C19Q target sites. Figure S2. Negative correlation of H3K9 trimethylation and active transcription. The H3K9me3 state and RNA levels of 30-40 Mb regions in chromosome 1-6 were shown in A-F respectively. The ChIP-seq of H3K9me3 and RNA-seq were performed on U2OS-EpiGo-Control cell lines. Figure S3. The effect of target distance on gene expression. (A) Plot showing the relationship between distance (CRISPR to the nearest TSS) and gene expression fold change. (B) Plot showing the relationship between distance (CRISPR to the nearest TSS) and promoter H3K4me3 enrichment change. (C) Plot showing the relationship between distance (CRISPR to the nearest TSS) and promoter H3K27ac enrichment change. [file 13059_2020_2201_MOESM1_ESM.zip › Additional file 1/Figure S2.pdf]

**A**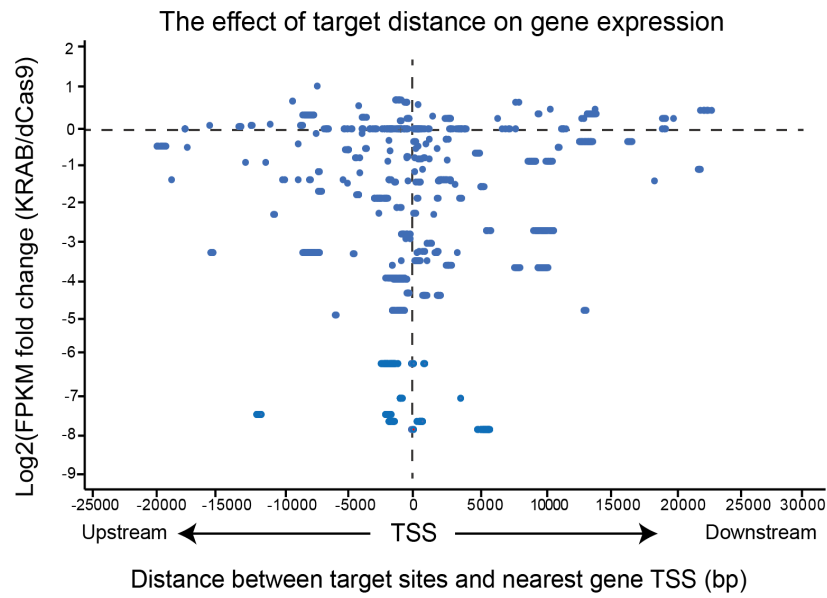**B**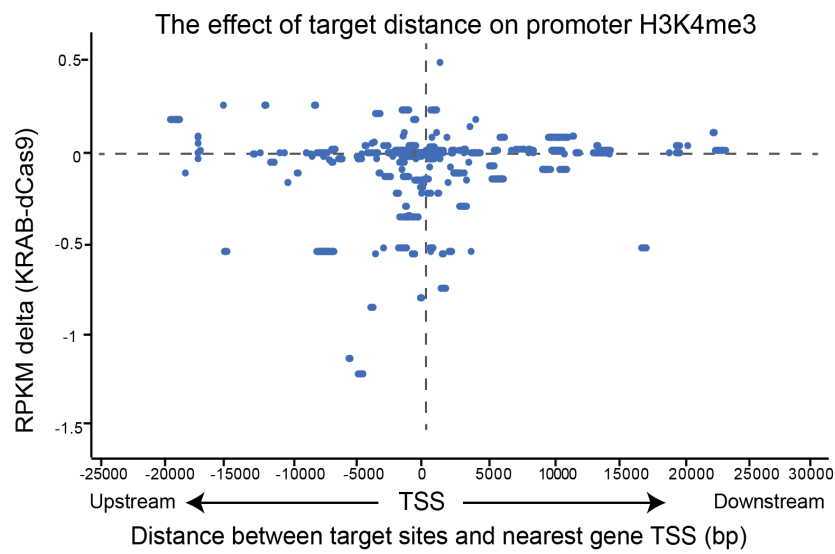**C**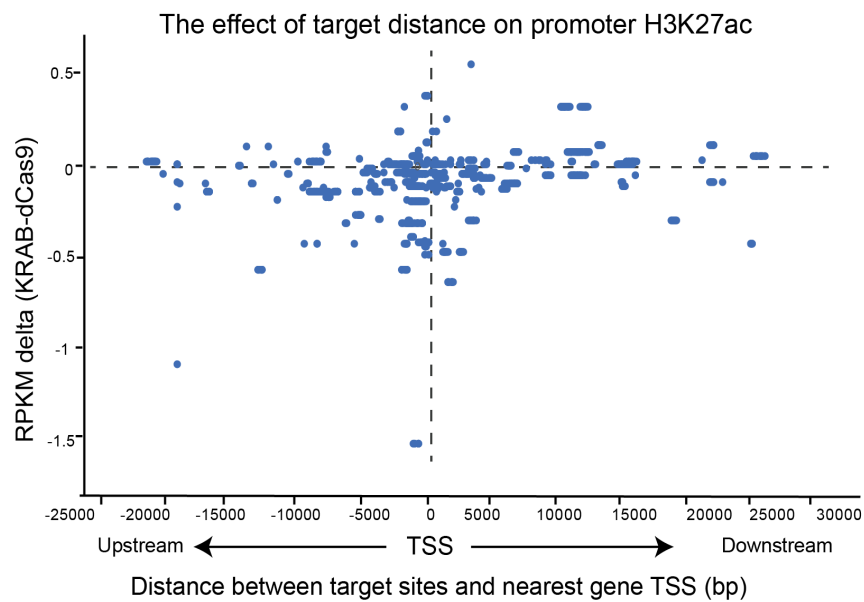

Supplement: Supplementary file 1 — Additional file 1: Figure S1. H3K9 trimethylation of C19Q regions mediated by the EpiGo-KRAB system. (A) H3K9me3 states of C19Q region in the cell lines of U2OS wildtype (WT), U2OS-HP1α-HaloTag (HP1α), EpiGo-Control (dCas9) or EpiGo-KRAB (dCas9-KRAB). ChIP-seq of H3K9me3 was performed in these four cell lines. C19Q repeats shown the C19Q target sites. (B) C19Q-1 region is chosen to show the difference of H3K9me3 state between U2OS wildtype (WT), U2OS-HP1α-HaloTag (HP1α), EpiGo-Control (dCas9) or EpiGo-KRAB (dCas9-KRAB) cell lines. C19Q repeats shown the C19Q target sites. Figure S2. Negative correlation of H3K9 trimethylation and active transcription. The H3K9me3 state and RNA levels of 30-40 Mb regions in chromosome 1-6 were shown in A-F respectively. The ChIP-seq of H3K9me3 and RNA-seq were performed on U2OS-EpiGo-Control cell lines. Figure S3. The effect of target distance on gene expression. (A) Plot showing the relationship between distance (CRISPR to the nearest TSS) and gene expression fold change. (B) Plot showing the relationship between distance (CRISPR to the nearest TSS) and promoter H3K4me3 enrichment change. (C) Plot showing the relationship between distance (CRISPR to the nearest TSS) and promoter H3K27ac enrichment change. [file 13059_2020_2201_MOESM1_ESM.zip › Additional file 1/Figure S3.pdf]
