# Supplementary material for: Simultaneous epigenetic perturbation and genome imaging reveal distinct roles of H3K9me3 in chromatin architecture and transcription
Source: Genome Biol. 2020 Dec 8;21:296. doi: 10.1186/s13059-020-02201-1 (PMC7722448; doi:10.1186/s13059-020-02201-1)
Supplement: Supplementary file 7 — Additional file 7. Review history. [file 13059_2020_2201_MOESM7_ESM.docx]

Review History

**First round of review**

**Reviewer 1**

**Are you able to assess all statistics in the manuscript, including the appropriateness of statistical tests used?** Yes, and I have assessed the statistics in my report.

**Comments to author:**

This manuscript reports the EpiGo method, which can simultaneously modulate chromatin state and visualize chromatin organization in living cells. The manuscript combines these two established CRISPR/dCas9 techniques so that H3K9Me3-induced chromatin reorganizations can be observed. It is useful for the study of connections between domains of histone markers and spatial organization of genomic elements. This manuscript is appropriate to the audience of Genome Biology.

The major concern from this reviewer is the lack of statistical rigor in some parts of this manuscript:

1. Figures 2C & F: An objective definition of C19Q "cluster" must be provided.

2. Figures 2 C-F, Figure 4 C-E: Number of cells analyzed in each experiment and "n" must be defined. The meaning of the error bar at 100% must also be explained.

3. Figure S2 indeed show colocalization of H3K9me3 and HP1apha, but not for all H3K9me3 or HP1alpha clusters (as can be seen in panel A). Therefore, the claim that "H3K9me3 surrounded the HP1alpha condensates" in the last paragraph on page 5 require statistical analysis.

4. The claim on page 6 that "the mobility of C19Q loci dramatically decreased in EpiGo-KRAB cell lines (Fig. S3)" cannot be supported by a single time series recording but require statistical analysis of a sufficient number of samples.

5. The claim on page 8 that "Region 1 collapsed into one focal spot, but now became associated with HP1alpha" require quantitative definition of "association" (because no strict colocalization can be seen in Figure 4) and statistical test to support the reduction of "association" (some level of "association" can be seen in the leftmost column of Figure 4B).

**Reviewer 2**

**Are you able to assess all statistics in the manuscript, including the appropriateness of statistical tests used?** Yes, and I have assessed the statistics in my report.

**Comments to author:**

This manuscript demonstrates a technique termed EpiGo-KRAB to induce chromatin architecture changes through CRISPR mediated epigenetic mark deposition, and to simultaneously visualize the changes through fluorescence imaging of the targeted genomic regions with the same CRISPR system. EpiGo-KRAB can successfully induce heterochromatin-like domain formation as revealed by both imaging and Hi-C. The domain formation relies on H3K9me3 methyltransferase SETDB1, only represses transcription of a minority of genes, spreads relatively continuously in inactive regions but sporadically in active regions. This is a useful technique to the field of genome biology and cell biology. The design, execution and presentation of the research is of high quality. It is particularly interesting to see that significant changes in the chromatin region clustering induced by EpiGo-KRAB is not associated with strong changes in gene expression in an active region. The tool can be helpful to further investigate this apparent decoupling between chromatin architecture and gene expression, or additional conditions necessary for heterochromatin domains to really functionally establish. I support the publication of this manuscript in Genome Biology, if the authors can address the following comments:

Major comments:
Does the distance/orientation of the CRISPR target to the promoter affect the efficiency of H3K4me3 and H3K27ac removal at the promoter and the gene repression? The authors stated that the top five silenced genes all have ectopic H3K9me3 at the promoter. A more systematic analysis of the promoter-to-target distance for all genes is missing here.

It is somewhat surprising that the dCas9-KRAB did not lead to transcription repression in many cases in this work since this fusion was introduced as a CRISPR repressor, although it is known that dCas9-KRAB is less efficient in gene repression than dCas9-KRAB-MeCP2 ("An enhanced CRISPR repressor for targeted mammalian gene regulation" by Church lab). It would be great to know: When repression does occur with a more efficient repressor, whether chromatin folding further differ from the state shown in the current manuscript.

In the Hi-C experiment, does C19Q-KRAB induce chromatin compartmentalization changes at regions other than the C19Q region. It seems there are copies of this targeted repeat sequence elsewhere on Chr19 based on Fig. 1a. Is the presence of one targeted repeat sequence in an open region sufficient to convert that region to the closed compartment? If certain density of the target repeat is required, is it possible to quantify that threshold density? This information will guide future application of the technique for targeted manipulation of chromatin architecture.

"Region 3, which is extensively modified by H3K9me3 in EpiGo-KRAB cells, has one focal spot and is associated with HP1aphla (Fig. 4B-4C)." It should be "EpiGo-control cells" here. And contrary to the claim, the association with HP1alpha does not seem to be stronger than Region 1 based on the sample images (Fig. 4B bottom right panel versus bottom left panel).

Minor comments:
In the description of fig.2, C19Q foci is said to decorate the surface of HP1alpha condensates in EpiGo-KRAB. However, from the images it seems C19Q foci and HP1alpha foci are partially overlapping and mostly next to each other. Is it equally legitimate to say that HP1apha condensates decorate the surface of C19Q foci?

I think the authors used the term "MERFISH" to describe the chromatin tracing technique introduced in citation 12. Although MERFISH was developed by the same group as citation 12, it is more accurate to refer to the multiplexed DNA FISH technique as "chromatin tracing" as the group does, since the DNA application did not involve the error robust (ER) coding as in the MERFISH technique.
The authors wrote: "OligoSTORM, MERFISH, Hi-M or ORCA has been applied to trace DNA folding [12-18] in fixed cells." Citation 13 does not trace DNA folding but visualizes single or pairs of domains. Citation 18 is all about live cells.

In the abstract the authors wrote: "Ectopic H3K9me3 appears to spread in inactive regions but is largely restricted to transcriptional initiation sites in active regions." Does the authors mean "restricted FROM", since "the spreading frequently stops at the transcriptional initiation sites in active regions"?

**Authors’ responses to review**

**GENERAL RESPONSES**

We deeply appreciate the reviewers for their valuable comments and suggestions. Here we include a letter to address the remaining issues raised for the revised manuscript.

**POINT-BY-POINT RESPONSES**

**Reviewer #1:**

This manuscript reports the EpiGo method, which can simultaneously modulate chromatin state and visualize chromatin organization in living cells. The manuscript combines these two established CRISPR/dCas9 techniques so that H3K9Me3-induced chromatin reorganizations can be observed. It is useful for the study of connections between domains of histone markers and spatial organization of genomic elements. This manuscript is appropriate to the audience of Genome Biology.

The major concern from this reviewer is the lack of statistical rigor in some parts of this manuscript:

1. Figures 2C & F: An objective definition of C19Q "cluster" must be provided.

We thanks the reviewer for the valuable comments. We added the definition of C19Q "cluster" in the figure legend. C19Q "cluster" was defined as that all the C19Q loci were confined within the size of 1 cm in each dimension.

2. Figures 2 C-F, Figure 4 C-E: Number of cells analyzed in each experiment and "n" must be defined. The meaning of the error bar at 100% must also be explained.

In Figure 2C and Figure 4B (now Figure 7B), “n” is the C19Q labeling positive cells, which has been defined in the figure legend. The error bars (including at 100%) are standard deviation (SD) from three independent experiments. We clarified this information in figure legends.

3. Figure S2 indeed show colocalization of H3K9me3 and HP1apha, but not for all H3K9me3 or HP1alpha clusters (as can be seen in panel A). Therefore, the claim that "H3K9me3 surrounded the HP1alpha condensates" in the last paragraph on page 5 require statistical analysis.

We agreed that not all HP1alpha condensates and H3K9me3 were colocalized from 3D-SIM in Figure S2A (now in the new Figure 4C). There are 80% of HP1alpha condensates were surrounded or decorated by H3K9me3 based on our counting of HP1alpha condensates in 20 cells.

We further quantitatively compared the colocalization between H3K9me3 and HP1apha by the degree of overlapping from widefield fluorescence microscopy and 3D-SIM. As shown in **Figure 4A and 4B in the revision and below**, there are extensive overlapping between H3K9me3 and HP1alpha by conventional widefiled microscopy, while HP1alpha condensates were surrounded or decorated by H3K9me3 with less degree of overlapping by structured illumination microscopy (3D-SIM) (**Fig. 4C-4D, also shown below**). Statistic analysis showed H3K9me3 and HP1alpha areas showed ~ 52% overlap in images from widefield microscopy, which decrease to 25% when measured by 3D-SIM (**Fig. 4E, also shown below**).

**Figure 4. Colocalization of H3K9me3 and HP1α in U2OS cells. A,** Imaging of H3K9me3 and HP1α colocalization by widefield fluorescence microscopy. H3K9me3 puncta (green) were detected by H3K9me3 antibody and HP1α (red) was tagged by HaloTag. **B,** Linescan of the vertical line. linescan shows, from top to bottom, the intensities (arbitrary units) of H3K9me3 (green) and HP1α (red) along the line indicated in (A). **C,** Imaging of H3K9me3 and HP1α colocalization by widefield fluorescence microscopy 3D-SIM. The image processing details are the same as described in (A). **D,**Linescan of the vertical line. The image processing details are the same as described in (B). **E,**Percentage of overlapping area between HP1α and H3K9me3 from images captured by widefield fluorescence microscopy and 3D-SIM. The outer edges of the box are the 25th and 75th percentiles. The whiskers extend to the minimum and maximum values. n=20 cells in each experiment.

4. The claim on page 6 that "the mobility of C19Q loci dramatically decreased in EpiGo-KRAB cell lines (Fig. S3)" cannot be supported by a single time series recording but require statistical analysis of a sufficient number of samples.

We thank the reviewer for the suggestion. As shown in the statistical data for Fig. S3 (now **Figure 3C and 3F, shown below**), the displacements (distances from the start position) over time in EpiGo-dCas9 control cells change more dramatically than in the EpiGo-KRAB cells.

**Fig. 3. Tracking the Dynamics of C19Q loci in EpiGo-KRAB cells.** **C,** Displacement of C19Q loci in EpiGo-Control cell lines. Distance to the start point of each time point was measured and plotted. n=20 cells. **F,** Displacement distance of C19Q loci in EpiGo-KRAB cell lines. Distance to the start point of each time point was measured and plotted. n=17 cells.

5. The claim on page 8 that "Region 1 collapsed into one focal spot, but now became associated with HP1alpha" require quantitative definition of "association" (because no strict colocalization can be seen in Figure 4) and statistical test to support the reduction of "association" (some level of "association" can be seen in the leftmost column of Figure 4B).

We thank the reviewer for pointing this out. We redefined the association or non-association by overlapping (regardless of the degrees) or non-overlapping between Regions and HP1alpha condensates. As shown in the statistical data for Figure 4 (now **Figure 7D and 7G, also shown below**), the percentages of foci association with HP1alpha in Region 1 and Region 2 are much higher in EpiGo-KRAB cells than in EpiGo-Control cells.

**Fig. 7. EpiGo-KRAB induced de novo heterochromatin-like domain formation.**

**D,** Percentage of foci association with HP1α in EpiGo-Control cell lines. The association or non-association is defined by overlapping (regardless of the degrees) or non-overlapping between foci and HP1α condensates. n=16, 16 and 12 cells from left to right respectively. **G,** Percentage of foci association with HP1α in EpiGo-KRAB cell lines. The association or non-association is defined by overlapping (regardless of the degrees) or non-overlapping between foci and HP1α condensates. n=10, 15 and 12 cells from left to right respectively.

**Reviewer #2:**

This manuscript demonstrates a technique termed EpiGo-KRAB to induce chromatin architecture changes through CRISPR mediated epigenetic mark deposition, and to simultaneously visualize the changes through fluorescence imaging of the targeted genomic regions with the same CRISPR system. EpiGo-KRAB can successfully induce heterochromatin-like domain formation as revealed by both imaging and Hi-C. The domain formation relies on H3K9me3 methyltransferase SETDB1, only represses transcription of a minority of genes, spreads relatively continuously in inactive regions but sporadically in active regions. This is a useful technique to the field of genome biology and cell biology. The design, execution and presentation of the research is of high quality. It is particularly interesting to see that significant changes in the chromatin region clustering induced by EpiGo-KRAB is not associated with strong changes in gene expression in an active region. The tool can be helpful to further investigate this apparent decoupling between chromatin architecture and gene expression, or additional conditions necessary for heterochromatin domains to really functionally establish. I support the publication of this manuscript in Genome Biology, if the authors can address the following comments:

**Major comments:**

**Comment 1:** Does the distance/orientation of the CRISPR target to the promoter affect the efficiency of H3K4me3 and H3K27ac removal at the promoter and the gene repression? The authors stated that the top five silenced genes all have ectopic H3K9me3 at the promoter. A more systematic analysis of the promoter-to-target distance for all genes is missing here.

**Response**: We thank the reviewer for the valuable comments. We have examined the relationship between the distance of CRISPR targets to gene promoters and their repression efficacy (The new **Figure S3** **and showing below**). The result suggested that when closer the dCas9 target is to its nearby promoter, the more likely the respective gene would be repressed. Such observation is also confirmed by the analyses of promoter H3K4me3 and H3K27ac changes. By contrast, the orientation does not have apparent effects on gene repression.

**Figure S3. The effect of target distance on gene expression**. **A,** Plot showing the relationship between distance (CRISPR to the nearest TSS) and gene expression fold change. **B,** Plot showing the relationship between distance (CRISPR to the nearest TSS) and promoter H3K4me3 enrichment change. **C,** Plot showing the relationship between distance (CRISPR to the nearest TSS) and promoter H3K27ac enrichment change.

**Comment 2:** It is somewhat surprising that the dCas9-KRAB did not lead to transcription repression in many cases in this work since this fusion was introduced as a CRISPR repressor, although it is known that dCas9-KRAB is less efficient in gene repression than dCas9-KRAB-MeCP2 ("An enhanced CRISPR repressor for targeted mammalian gene regulation" by Church lab). It would be great to know: When repression does occur with a more efficient repressor, whether chromatin folding further differ from the state shown in the current manuscript.

**Response**:
Thanks for the valuble suggestion. As we can see from Figure 5E in the revision, the transcription repression are usually observed when dCas9-KRAB is targeted directly to the promoters, while those targeted outside of promoters are not associated with repression. It is possible that dCas9-KRAB-MeCP2 might induce even higher chromatin compaction than dCas9-KRAB. We tried to focus on H3K9me3 in this story, and we are concerned that the introduction of MeCP2 may bring a confounding factor - DNA methylation (which MeCP2 binds). Therefore, we hope to study the effect of this dCas9 repressor in future studies.

**Comment 3:** In the Hi-C experiment, does C19Q-KRAB induce chromatin compartmentalization changes at regions other than the C19Q region. It seems there are copies of this targeted repeat sequence elsewhere on Chr19 based on Fig. 1a.

**Response**: We thank the reviewer for pointing this out. The previous figure is somewhat misleading when showing a zoomed-out version of C19Q. We confirm that all the repeat sequences targeted by C19Q-KRAB in Fig. 1A are in the C19Q region. We apologize for the confusion and we have now revised **Figure 1A**.

**Comment 4:** Is the presence of one targeted repeat sequence in an open region sufficient to convert that region to the closed compartment? If certain density of the target repeat is required, is it possible to quantify that threshold density? This information will guide future application of the technique for targeted manipulation of chromatin architecture.

**Response**: We thank the reviewer for raising these important questions. We have now conducted in-depth analysis of the relationship between dCas9-KRAB targeting and compartment conversion, which have revealed interesting findings as described below.

We first asked what would be the threshold of dcas9-KRAB target density to achieve a compartment A-to-B switch. A close examination revealed that out of 49 compartment A regions (100kb bin) with target sequences in dCas9 cells, surprisingly only 4 of them were converted to compartment B in KRAB cells (A-to-B), while the rest 45 remained as compartment A (A-to-A), raising the possibility that the target density may need to exeed a threshold to flip a compartment A to B. Indeed, we found that the average target densities (per 100kb bin) in A-to-B regions (n=10, 49,18, 20, respectively, see red box in **Figure 9A-9B**) are much higher than A-to-A regions (**Figure 9C**), supporting the notion that high target density is needed to achieve chromatin organization alterations.

Nevertheless, the dense targeting of dCas9-KRAB is clearly not a simple rule, as some A-to-A regions also show high target density (**Figure 9C**). One A-to-A example can be seen in **Figure 9A-9B** which show dense presence of dCas9-KRAB targets (green box). Notably, these regions appear to enrich for active genes (**Figure 9A-9B**), raising the possibility that additional factors may be also involved in compartment switch. Notably, our recent study showed that during SCNT, many compartments A and B remain constant between mouse donor cells and SCNT embryos, and they preferentially have the highest and lowest gene densities respectively[1]. By contrast, switched compartment regions tend to have intermediate gene density. We now confirmed such results in additional mouse cells (MEF vs. cumulus cells[1, 2] and human cells (hESC vs trophectoderm[3]) (**Figure 9D**). Similar results were also reported for lamina-associated domains (LADs) which are closely associated with compartments A/B[4]. Regions with high and low gene density tend to be constant LADs (cLADs) and constant inter-LADs, while variable LADs (vLADs) preferentially show intermediate gene density and frequently reside at the boundary of LADs and iLADs[5]. Our analyses revealed that compared to the whole genome, chr19 has unusually high levels of gene density, including both A-to-B and A-to-A regions (**Figure 9E**), suggesting that these regions may be naturally resistant to A-to-B switches. In addition, A-to-A regions show higher gene expression and histone acetylation levels than A-to-B regions, supporting that A-to-A regions are more likely to be stable compartment A (**Figure 9F**). Finally, it was reported that variable LADs (vLADs) frequently reside at the boundary of LADs and iLADs[5]. Similarly, we found that A-to-B regions are preferentially located near A/B boundaries (**Figure 9G**, see example in **Figure 9A-B**, red box).

In sum, our new analysis show that compartment switch is likely regulated by multiple factors including dcas9-KRAB binding, closeness to A-B borders, and gene density. Compartment A with targeted sequences in C19q show particularly high-gene density in most regions (**Figure 9E**), suggesting that these regions may be naturally more resistant to A-to-B switch, unless they have abundant sgRNA targets and/or are near compartment A-B boundaries. We thank the reviewer’s insightful questions that led to these new findings.

**Figure 9. Compartment switch is linked to multi-factors**. **A-B,** Hi-C heatmap showing the compartmentalization of the C19Q region in EpiGo-Control (dCas9) (A) and EpiGo-KRAB (B) cells (chr19: 40,000,000-50,000,000). H3K9me3, RNA-seq and PC1 tracks are also shown. Red box, A-to-B switched regions upon dCas9-KRAB induction. Green box, A-to-A regions upon dCas9-KRAB induction. **C,** Boxplots showing the dCas9-KRAB target sequence density for A-to-A and A-to-B regions in dCas9 and dCas9-KRAB cells. Only compartment A regions with target sequences were selected for analysis. **D,** Left, boxplots showing the gene density in each class for mouse cell lines (MEF A-to-CC B, MEF B-to-CC A, MEF A-to-CC B, MEF B-to-CC A). Right, boxplots showing the gene density in each class for human cell lines (hES A-to-TB B, hES B-to-TB A, hES A-to-TB B, hES B-to-TB A). MEF, mouse embryonic fibroblast; CC, cumulus cells; hES, human embryonic stem cells; TB, trophectoderm. Hi-C data were obtained from GSE52457 and GSE139430. **E,** Boxplots showing the gene density in each class. Whole genome compartment A/B and all chr19 compartment A/B in dCas9 cells are similarly analyzed and shown as control. **F,** Left, boxplots showing the gene expression level in each class. Right, boxplots showing the H3K27ac enrichment at promoters for genes located in each class. Whole genome compartment A/B and all chr19 compartment A/B in dCas9 cells were are similarly analyzed and shown as controls. **G,** Boxplots showing the average distance towards A/B borders in each class. Whole genome compartments A/B and all chr19 compartments A/B in dCas9 cells were used as control. All A-to-B regions in chr19q arm with dCas9-KRAB targets were similarly analyzed and shown.

**Comment 4:** "Region 3, which is extensively modified by H3K9me3 in EpiGo-KRAB cells, has one focal spot and is associated with HP1aphla (Fig. 4B-4C)." It should be "EpiGo-control cells" here. And contrary to the claim, the association with HP1alpha does not seem to be stronger than Region 1 based on the sample images (Fig. 4B bottom right panel versus bottom left panel).

Thanks the reviewer to point out the error and we have now fixed it in the main text. We also agreed that the degree of overlapping between Region 3 and HP1alpha might not be higher to some loci of Region 1 and HP1alpha. As shown in Fig. 4B bottom panel (now Fig. 7B), Region 1 has multiple foci with one spot associating with HP1alpha, while Region 3 has shown only one spot associating with HP1alpha almost in every cell. We quantified the percentage of foci associated with HP1alpha in EpiGo-control and EpiGo-KRAB cells in **Figure 7D, 7G and the result is shown as below**.

**Fig. 7. EpiGo-KRAB induced de novo heterochromatin-like domain formation.**

**D,** Percentage of foci association with HP1α in EpiGo-Control cell lines. The association or non-association is defined by overlapping (regardless of the degrees) or non-overlapping between foci and HP1α condensates. n=16, 16 and 12 cells from left to right respectively. **G,** Percentage of foci association with HP1α in EpiGo-KRAB cell lines. The association or non-association is defined by overlapping (regardless of the degrees) or non-overlapping between foci and HP1α condensates. n=10, 15 and 12 cells from left to right respectively.

**Minor comments:**

**Comment 5:** In the description of fig.2, C19Q foci is said to decorate the surface of HP1alpha condensates in EpiGo-KRAB. However, from the images it seems C19Q foci and HP1alpha foci are partially overlapping and mostly next to each other. Is it equally legitimate to say that HP1apha condensates decorate the surface of C19Q foci?

We agreed that it was hard to distinguish whether HP1alpha decorates C19Q or the other way around in these images. Nevertheless, based on the 3D-SIM data showed in **Figure 4 in the revision**, H3K9me3 was at the surface on HP1alpha condensates, which mades us believe that chromatin DNA including C19Q loci should decorate on the surface of HP1alpha condensates. We now revised our statement in the manuscript (page 6) to better reflect this reasoning.

**Comment 6:** I think the authors used the term "MERFISH" to describe the chromatin tracing technique introduced in citation 12. Although MERFISH was developed by the same group as citation 12, it is more accurate to refer to the multiplexed DNA FISH technique as "chromatin tracing" as the group does, since the DNA application did not involve the error robust (ER) coding as in the MERFISH technique.

Thanks the reviewer for helping us to use these terms more accurately. We corrected them in the main text.

**Comment 7:** The authors wrote: "OligoSTORM, MERFISH, Hi-M or ORCA has been applied to trace DNA folding [12-18] in fixed cells." Citation 13 does not trace DNA folding but visualizes single or pairs of domains. Citation 18 is all about live cells.

Thanks the reviewer for pointing this out. We corrected the references.

**Comment 8:** In the abstract the authors wrote: "Ectopic H3K9me3 appears to spread in inactive regions but is largely restricted to transcriptional initiation sites in active regions." Do the authors mean "restricted FROM", since "the spreading frequently stops at the transcriptional initiation sites in active regions"?

Yes, we do mean that H3K9me3 spreading is restricted FROM transcriptional initiation sites. We have corrected this in the abstract and we thank the reviewer for pointing this out.

**Reference**

1.	Zhang K, Wu DY, Zheng H, Wang Y, Sun QR, Liu X, Wang LY, Xiong WJ, Wang Q, Rhodes JDP, et al: Analysis of Genome Architecture during SCNT Reveals a Role of Cohesin in Impeding Minor ZGA. *Mol Cell* 2020, **79**:234-250 e239.

2.	Barutcu AR, Maass PG, Lewandowski JP, Weiner CL, Rinn JL: A TAD boundary is preserved upon deletion of the CTCF-rich Firre locus. *Nat Commun* 2018, **9**:1444.

3.	Dixon JR, Jung I, Selvaraj S, Shen Y, Antosiewicz-Bourget JE, Lee AY, Ye Z, Kim A, Rajagopal N, Xie W, et al: Chromatin architecture reorganization during stem cell differentiation. *Nature* 2015, **518**:331-336.

4.	van Steensel B, Belmont AS: Lamina-Associated Domains: Links with Chromosome Architecture, Heterochromatin, and Gene Repression. *Cell* 2017, **169**:780-791.

5.	Meuleman W, Peric-Hupkes D, Kind J, Beaudry JB, Pagie L, Kellis M, Reinders M, Wessels L, van Steensel B: Constitutive nuclear lamina-genome interactions are highly conserved and associated with A/T-rich sequence. *Genome Res* 2013, **23**:270-280.

**Second round of review**

**Reviewer 1**

The revised manuscript has satisfactorily addressed previous concerns regarding statistics and rigor from this reviewer.

**Reviewer 2**

The authors have largely addressed my comments. However there are three remaining/new issues that need to be addressed:
1. In the revision, the authors wrote “C19Q "cluster" was defined as that all the C19Q loci were confined within the size of 1 cm in each dimension.” Cm is either a wrong unit, or the authors were measuring the images. Please use the right unit that reflects the true physical size inside of the cell nucleus.
2. All the new box plots in Figure 9 lack statistical testing. For example, the authors were trying to conclude that in D, the AA values are higher than AB/ BA values, which are further higher than BB values. Without statistical tests the box plots alone are not convincing, since the medians shown in a lot of these categories are the same in these plots.
3. The authors still argue that “based on the 3D-SIM data showed in Figure 4 in the revision, H3K9me3 was at the surface on HP1alpha condensates”, while I still fail to see that in the figure.  Based on the line profiles in B and D of Figure 4, the H3K9me3 peaks and HP1alpha peaks are slight shifted, meaning the condensates are next to each other. If this is what the author meant by saying “H3K9me3 was at the surface on HP1alpha condensates”, one can equally say “HP1apha was at the surface on H3K9me3 chromatin condensates”.  If the authors were trying to argue that H3K9me3 surrounds HP1aphla condensates, in the line profiles there should be pairs of green peaks on both sides of the each red peak, and I do not see that.

**Authors’ responses to review AND EDITORIAL COMMENTS**

**GENERAL RESPONSES**

We Addressed reviewer #2 questions below. We added the dataset information and reformatted the manuscript according to editorial guidelines.

**POINT-BY-POINT RESPONSES**

Reviewer #2: The authors have largely addressed my comments. However there are three remaining/new issues that need to be addressed:

1. In the revision, the authors wrote “C19Q "cluster" was defined as that all the C19Q loci were confined within the size of 1 cm in each dimension.” Cm is either a wrong unit, or the authors were measuring the images. Please use the right unit that reflects the true physical size inside of the cell nucleus.

Sorry for the error and confusion. It should be 1 um, not 1 cm. We have corrected in the legend of Figure 2C.

2. All the new box plots in Figure 9 lack statistical testing. For example, the authors were trying to conclude that in D, the AA values are higher than AB/ BA values, which are further higher than BB values. Without statistical tests the box plots alone are not convincing, since the medians shown in a lot of these categories are the same in these plots.

We added statistical testing for the new box plots in Figure 9.

3. The authors still argue that “based on the 3D-SIM data showed in Figure 4 in the revision, H3K9me3 was at the surface on HP1alpha condensates”, while I still fail to see that in the figure.  Based on the line profiles in B and D of Figure 4, the H3K9me3 peaks and HP1alpha peaks are slight shifted, meaning the condensates are next to each other. If this is what the author meant by saying “H3K9me3 was at the surface on HP1alpha condensates”, one can equally say “HP1apha was at the surface on H3K9me3 chromatin condensates”.  If the authors were trying to argue that H3K9me3 surrounds HP1aphla condensates, in the line profiles there should be pairs of green peaks on both sides of the each red peak, and I do not see that.

We have changed “H3K9me3 was at the surface on HP1alpha condensates” to be “H3K9me3 was adjacent to HP1alpha condensates” in the main text.
